# Building capacity to use and undertake research in health organisations: a survey of training needs and priorities among staff

**DOI:** 10.1136/bmjopen-2016-012557

**Published:** 2016-12-07

**Authors:** Helen Barratt, Naomi J Fulop

**Affiliations:** Department of Applied Health Research, University College London, London, UK

**Keywords:** Knowledge translation, Evidence use, Research participation, Training

## Abstract

**Objectives:**

Efforts to improve healthcare and population health depend partly on the ability of health organisations to use research knowledge and participate in its production. We report the findings of a survey conducted to prioritise training needs among healthcare and public health staff, in relation to the production and implementation of research, across an applied health research collaboration.

**Design:**

A questionnaire survey using a validated tool, the Hennessy-Hicks Training Needs Assessment Questionnaire. Participants rated 25 tasks on a five-point scale with regard to both their confidence in performing the task, and its importance to their role.

**Setting:**

A questionnaire weblink was distributed to a convenience sample of 35 healthcare and public health organisations in London and South East England, with a request that they cascade the information to relevant staff.

**Participants:**

203 individuals responded, from 20 healthcare and public health organisations.

**Interventions:**

None.

**Outcome measures:**

Training needs were identified by comparing median importance and performance scores for each task. Individuals were also invited to describe up to three priority areas in which they require training.

**Results:**

Across the study sample, evaluation; teaching; making do with limited resources; coping with change and managing competing demands were identified as key tasks. Assessing the relevance of research and learning about new developments were the most relevant research-related tasks. Participants’ training priorities included evaluation; finding, appraising and applying research evidence; and data analysis. Key barriers to involvement included time and resources, as well as a lack of institutional support for undertaking research.

**Conclusions:**

We identify areas in which healthcare and public health professionals may benefit from support to facilitate their involvement in and use of applied health research. We also describe barriers to participation and differing perceptions of research between professional groups.

**Strengths and limitations of this study**

- Our study is the first of its kind to be conducted in England and extends the existing literature exploring research use and participation in specific groups by examining self-identified opportunities to improve research use and comparing across professional groups.
- The findings may be relevant to others looking to establish research training programmes, because we received responses from staff in 20 separate organisations, including large teaching hospitals, small district general hospitals and public health organisations.
- Several professional groups were under-represented in our survey, and their perspectives warrant further exploration, for example, midwives and public health staff.
- Our sample size was relatively small, but the survey was conducted in the first few months of our 5-year research collaboration and it is encouraging to see that there was clear interest in using and applying research, right across the partnership.
- Results from a convenience sample are of unknown generalisability and staff who completed the survey are likely to be those who are most interested in the topic, but this sampling approach was appropriate for our purposes as we sought to prioritise our capability building efforts and reach those most interested in research.

## Background

The provision of high-quality, affordable, health services is a growing challenge in many developed countries. In England, for example, the National Health Service (NHS) 5-Year Forward View set out the case for major system change and new ways of working.^1^ Staff in healthcare and public health organisations have a key role to play in improving patient care and population health through the implementation and coproduction of applied health research (AHR).^2^ Emerging evidence suggests there is an association between the engagement of healthcare organisations in research and improvements in their overall performance.^3^ However, such organisations frequently fail to use research evidence to inform practice.^4^ ^5^ Similar findings have been reported globally, in primary and secondary care.^6^ In order to improve care, research findings therefore need to be better integrated into practice and organisational routines, alongside efforts to promote the coproduction of knowledge and build organisational absorptive capacity.^7^

Over the past 10–15 years, increasing attention has been paid to reducing the ‘know-do’ gap.^8^ Ellen *et al*^9^ set out a framework of possible organisational level activities that might be undertaken to facilitate access, dissemination, exchange and use of evidence within health organisations. The framework builds on earlier work by Lavis *et al*^10^ which classified approaches to communicating research to end users as push, pull or exchange efforts. It acknowledges that the path from research creation to usage may not be logical or linear, as well as the influence that context may have on decision-making. It includes four major domains of activity: (1) establishing a climate for research use; (2) research production efforts; (3) activities used to link research to action; and (4) evaluation.^9^ The third domain, activities to link research to action, consists of three parts. The first includes ‘push’ efforts, such as activities undertaken by researchers or intermediaries to disseminate research evidence. Second, ‘facilitating pull’ efforts aim to provide ‘easy access’ to research evidence, by ensuring that the appropriate infrastructure is in place to make the process straightforward for knowledge users (eg, IT systems, websites). Finally, ‘pull’ efforts seek to develop the personal capacity and capability of staff within health organisations. This includes, for example, training that focuses on the skills needed to find or appraise research evidence. Our specific focus in this paper is on this final component: training as a means of increasing participation in and use of AHR by health professionals.

The 2006 Cooksey Report highlighted the gap that exists in the UK between the conduct of research and its implementation.^11^ Subsequently, in 2007, a High Level Group on Clinical Effectiveness, chaired by Sir John Tooke, called on the health service to harness better the capacity of higher education to help address this problem. It recommended the development of new ‘academic health centres’ to encourage the conduct of relevant research and help embed a culture more receptive to change in the NHS.^12^ ^13^ Collaborations for Leadership in Applied Health Research and Care (CLAHRCs) were established in England^13^ to facilitate the coproduction of research by staff in the health service and public health departments, working together with academic researchers.^14^ ^15^ Funded by the National Institute for Health Research (NIHR), the first round of five CLAHRCs was established in 2009. Evaluation demonstrated that the first wave had differing capabilities with respect to reducing the ‘know-do’ gap, partly because of differing interpretations and enactments of their mission.^16^ However, success in this area will inevitably require a long-term, sustained focus on relationship building, resource allocation and, in some cases, culture change.^17^ The second wave of 13 CLAHRCs has been in operation across England since January 2014.

This article describes an exercise carried out to assess research training needs and priorities among healthcare and public health staff across England's largest CLAHRC, NIHR CLAHRC North Thames. Alongside our programme of research, we have established an Academy to build capacity and capability to coproduce research and apply its outputs in practice (http://www.clahrc-norththames.nihr.ac.uk/academy/). This exercise was conducted to inform the Academy's priorities and the development of a programme of activities, including short courses. Drawing on the framework proposed by Ellen *et al*,^9^ the aim of these activities is to increase participation in and use of AHR by health professionals, to better link research and action. This is the first such study conducted in the UK. Owing to the size and breadth of our partnership, our findings may be relevant to others seeking to establish similar programmes, addressing the training needs of a range of professional groups. They also contribute to a growing literature on research use, at a time when there is a need for evidence to support new ways of working in many healthcare systems.^1^ To date, much of this research has taken place outside of the UK and it has typically studied the different behaviours of specific health professions, such as nursing^18^ and allied health professionals.^19^ This literature suggests that training needs and priorities may differ between groups, but few previous studies have formally compared professions or examined self-identified opportunities to improve research use or participation.

## Methods

We used a self-administered online survey to explore research training needs across NIHR CLAHRC North Thames in June 2014. This approach was chosen to elicit a high volume of feedback in a short amount of time, from participants in geographically separated areas.^20^ The Hennessy-Hicks Training Needs Analysis Questionnaire is a validated tool, which offers a means of evaluating training requirements and prioritising education and development opportunities to meet local needs.^21^ It is tailored for use specifically with health teams and designed to be adapted, without compromising its validity and reliability.

### Study population

Launched in January 2014, and funded for 5 years, NIHR CLAHRC North Thames involves 55 partner organisations across North Central and East London, as well as parts of Bedfordshire, Essex and Hertfordshire. It covers a diverse population of over 6 million residents; 10% of the UK population. Partner organisations include higher education institutions, healthcare and public health organisations, as well as third sector organisations and industry partners. The intended audience for our programme of short courses is staff working in our 35 partner healthcare and public health organisations. These include 21 NHS provider organisations responsible for acute hospital services, mental health or community care (known as NHS Trusts); 8 organisations responsible for purchasing or commissioning care on behalf of patients in a designated geographical area (known as Clinical Commissioning Groups); and 8 local government departments, responsible for public health (known as Local Authorities).

### Administration

We used the web-based tool, Opinio, to collect the survey data from a convenience sample (https://www.ucl.ac.uk/isd/services/learning-teaching/elearning-staff/core-tools/opinio). An email was sent to key CLAHRC contacts in partner organisations, with a request that they disseminate the questionnaire weblink to staff electronically. The email explained that the survey was to inform the design of training opportunities for healthcare and public health staff, to increase their skills in using research evidence. It stated that we were keen to receive responses from staff with a range of backgrounds and experience, at all levels, from a range of groups including but not limited to clinicians; nurses and midwives; allied health professionals; managers and technical staff, such as laboratory workers. Reminder emails were sent 2 weeks later and the survey was live for 4 weeks in total.

### Questionnaire development

The survey questions were developed in line with the guidance set out in the questionnaire manual.^21^ The basic questionnaire comprises a list of 30 tasks, relating to a range of areas, including research, communication/teamwork, clinical tasks, administration and management. Each item is rated along a seven-point scale with respect to how important the task is to the respondent's job (Rating A); and how well the task is currently performed (Rating B). Comparing scores for self-assessed importance/performance provides an assessment of where the greatest training needs lie. The greater the difference in scores, the greater the training need. The questionnaire also facilitates comparison between the different tasks, such as research and administration.

The questionnaire is designed so that up to 25% of the original items (to a maximum of 8) may be swapped for items of the researcher's choice without compromising its psychometric properties. Another 10 items may be added in.^21^ The modification process involved two stages. First, we identified possible additional tasks from the literature on research use and participation by healthcare and public health staff, and through one to one interviews with staff from a range of backgrounds (n=7). We did this iteratively by sense checking new suggestions with subsequent interviewees. Before releasing the survey, we pilot tested it with eight staff from a range of professional backgrounds, drawn from across the CLAHRC. In the second stage of the modification process, we presented the proposed changes to these individuals and asked them to comment on whether the alternations appeared valid to them. Throughout the modification process, we considered design factors such as the quality of the questions, survey format and the way questions were presented.^22^ Pilot testers were provided with a copy of the draft questionnaire. As well as asking them about the proposed modifications to the list of tasks, we asked them whether the text of the questionnaire was clear and how realistic it was in the context of their current role. Overall, the tool was considered to be good, with clear instructions and of appropriate length. Through this process we made minor modifications to the tool including: adding in a definition of research; providing additional job categories in the section on demographic data; and clarifying the instructions for the importance/performance rating exercise. Pilot testers considered the list of tasks to be clear and comprehensible, acknowledging the challenges of compiling a list that would be of broad relevance across a range of different types of health organisation. Two testers suggested that we group together similar tasks. However, the questionnaire developers intended that the list should be organised randomly, so we opted to keep this approach to maintain the integrity of the tool.

Section 1 of the final survey included a list of 25 tasks, 13 of which were directly related to research. These are listed in table 1. In line with guidance about the use of the questionnaire, we retained 22/30 of the original survey items. In section 2, participants were invited to list up to three areas in which they felt they would benefit from training to better equip them either to conduct research or apply its findings in practice. We also collected basic demographic information, including professional group, age and gender.

**Table 1:** Research training needs by profession

	All respondents (n=151)			Allied health professionals (n=39)			Doctors (n=36)			Managers (n=22)			Nurses (n=27)		
	Importance to role (median score)	Current Performance (median score)	p Value	Importance to role (median score)	Current Performance (median score)	p Value	Importance to role (median score)	Current Performance (median score)	p Value	Importance to role (median score)	Current Performance (median score)	p Value	Importance to role (median score)	Current Performance (median score)	p Value
1. Handling routine data	6.00	5.00	0.03	5.00	5.00	0.63	6.00	6.00	1.00	6.00	5.50	0.18	7.00	6.00	<0.01
2. Critically evaluating published research	5.00	4.00	<0.01	5.00	4.00	0.01	6.00	4.50	<0.01	4.00	4.00	0.87	6.00	4.00	<0.01
3. Evaluating your organisation's performance	6.00	4.00	<0.01	6.00	4.00	<0.01	5.00	4.00	<0.01	7.00	5.00	**<0.01**	7.00	4.00	<0.01
4. Interpreting research findings	5.00	5.00	<0.01	5.00	4.00	<0.01	6.00	5.00	<0.01	5.00	4.00	0.88	6.00	4.00	<0.01
5. Applying research results to your own practice	6.00	4.00	<0.01	6.00	4.00	**<0.01**	6.00	5.00	<0.01	5.50	4.00	<0.01	6.00	4.00	<0.01
6. Identifying viable research topics	4.00	3.00	<0.01	4.00	3.00	0.02	4.50	3.00	0.02	3.50	4.00	0.48	4.50	3.50	<0.01
7. Introducing new ideas at work	6.00	5.00	<0.01	6.00	4.00	<0.01	6.00	4.50	<0.01	7.00	5.00	<0.01	7.00	5.00	<0.01
8. Accessing relevant research literature to inform your work	6.00	5.00	<0.01	6.00	4.00	<0.01	6.00	4.50	<0.01	6.00	4.50	<0.01	6.00	5.00	<0.01
9. Giving information about research to patients/the public	5.00	4.00	<0.01	5.00	4.00	<0.01	5.00	4.50	0.01	4.50	4.00	0.07	5.50	4.00	0.08
10. Statistically analysing your own research data	4.00	3.00	<0.01	4.00	3.00	0.04	5.00	3.00	<0.01	4.00	3.00	0.02	5.00	2.50	<0.01
11. Teaching colleagues and/or students	6.00	5.00	<0.01	7.00	5.00	<0.01	6.00	5.00	<0.01	6.50	5.00	**<0.01**	7.00	6.00	0.03
12. Managing multiple demands on your time	7.00	5.00	**<0.01**	7.00	5.00	**<0.01**	7.00	5.00	**<0.01**	7.00	5.00	**<0.01**	7.00	5.00	**<0.01**
13. Writing up the findings of research studies or audits	5.00	4.00	<0.01	5.00	4.00	0.02	5.00	4.00	<0.01	3.00	4.00	0.64	5.50	4.00	<0.01
14. Undertaking health promotion activities	5.00	4.00	<0.01	5.00	3.00	<0.01	5.00	3.00	<0.01	2.00	3.00	0.84	5.00	4.50	0.01
15. Making do with limited resources	6.00	5.00	<0.01	6.00	5.00	0.04	5.00	4.00	<0.01	6.00	5.00	<0.01	6.00	5.00	0.28
16. Assessing local healthcare needs	5.00	4.00	<0.01	4.00	3.00	<0.01	5.00	3.00	<0.01	5.00	3.00	<0.01	5.00	4.00	0.01
17. Collecting and collating relevant research	5.00	4.00	<0.01	4.50	3.00	<0.01	5.00	3.00	<0.01	5.00	4.00	0.27	5.00	4.00	0.09
18. Designing research studies	3.00	3.00	<0.01	3.00	3.00	0.01	4.50	2.50	<0.01	3.00	0.24	0.31	4.00	2.00	<0.01
19. Working as a member of a team doing research	4.00	4.00	0.03	4.00	3.00	0.14	5.00	4.00	0.35	3.00	3.00	0.34	6.00	5.00	0.06
20. Accessing resources to undertake research eg, money, information, equipment	4.00	2.00	<0.01	3.00	2.00	<0.01	5.00	2.00	<0.01	4.00	3.50	<0.01	4.50	3.50	<0.01
21. Undertaking administrative activities	5.00	5.00	0.15	5.00	5.00	0.98	5.00	4.00	<0.01	6.00	5.50	0.54	5.00	5.00	0.83
22. Personally coping with change in the health service	6.00	5.00	<0.01	6.00	5.00	<0.01	6.00	4.00	<0.01	6.00	5.00	0.02	6.00	4.00	<0.01
23. Securing time to undertake research	5.00	2.00	<0.01	4.00	2.00	<0.01	5.00	2.00	**<0.01**	4.00	3.00	0.04	5.00	2.00	**<0.01**
24. Learning about new research developments in your field	6.00	4.00	**<0.01**	6.00	4.00	**<0.01**	6.00	4.00	**<0.01**	6.00	4.00	<0.01	6.00	4.00	**<0.01**
25. Assessing the relevance of research to your organisation	5.00	4.00	**<0.01**	5.00	4.00	<0.01	5.00	4.00	<0.01	5.00	4.00	<0.01	5.00	4.00	<0.01

Numbers in bold and underlined represent the three most significant training needs for each profession.

### Analysis

We used Microsoft Excel to manage the survey data and analyse the data from section 1, comparing self-assessed importance and performance ratings for each task to identify training needs. Given that much of the existing literature focuses on individual professional groups in isolation, we analysed results for the whole sample, and also disaggregated the data to explore whether differences exist between the needs of different professional groups. To establish whether differences between the importance and performance scores given to each task were significant, and therefore represented a training need, Wilcoxon signed ranks tests were conducted in Microsoft Excel using the Real Statistics Resource Pack add in (http://www.real-statistics.com). The survey tool authors have advocated the use of parametric tests to analyse the data it generates.^21^ ^23^ ^24^ However, because one cannot necessarily assume that the intervals are equal between values in Likert-type scales, such as those used to rate performance and importance, we have opted to use a non-parametric approach.^25^ We carried out qualitative content analysis of free text data from section 2 of the questionnaire to identify research training priorities, using the systematic method set out by Mayring.^26^ Categories were derived iteratively using Mayring's step model of inductive category development. Within this, the researcher (HB) reviewed all the free text data in light of the research questions. Free text comments relating to similar topics (eg, training in research methods; using research in practice) were grouped together. From this, provisional categories were deduced and revised, with constant reference to the data. The reliability of the final categories was then checked by the research team, before quantitative aspects of the analysis (eg, frequency of the coded categories) were conducted by HB. Priorities were first identified for the whole sample, and then compared using the same categories to examine potential differences between professional groups.

### Ethics approval

Completion of the NHS Health Research Authority's decision tool indicated that NHS ethics approval was not required for our needs assessment.^27^ Local ethics approval was also not required because the study only involved the use of survey methods to collect non-sensitive, anonymous information from participants who were not defined as vulnerable.

## Results

In this section, we describe first the demographics of the survey respondents. We then go on to examine the research training needs identified by comparing importance and performance ratings for each task; the training priorities described by participants; and finally the barriers to research that were highlighted by respondents.

### Demographics

A total of 203 individuals completed at least one part of the questionnaire. 151 completed the rating exercise and 125 also described at least one research-related training need in the free text section.

**Figure 1:**
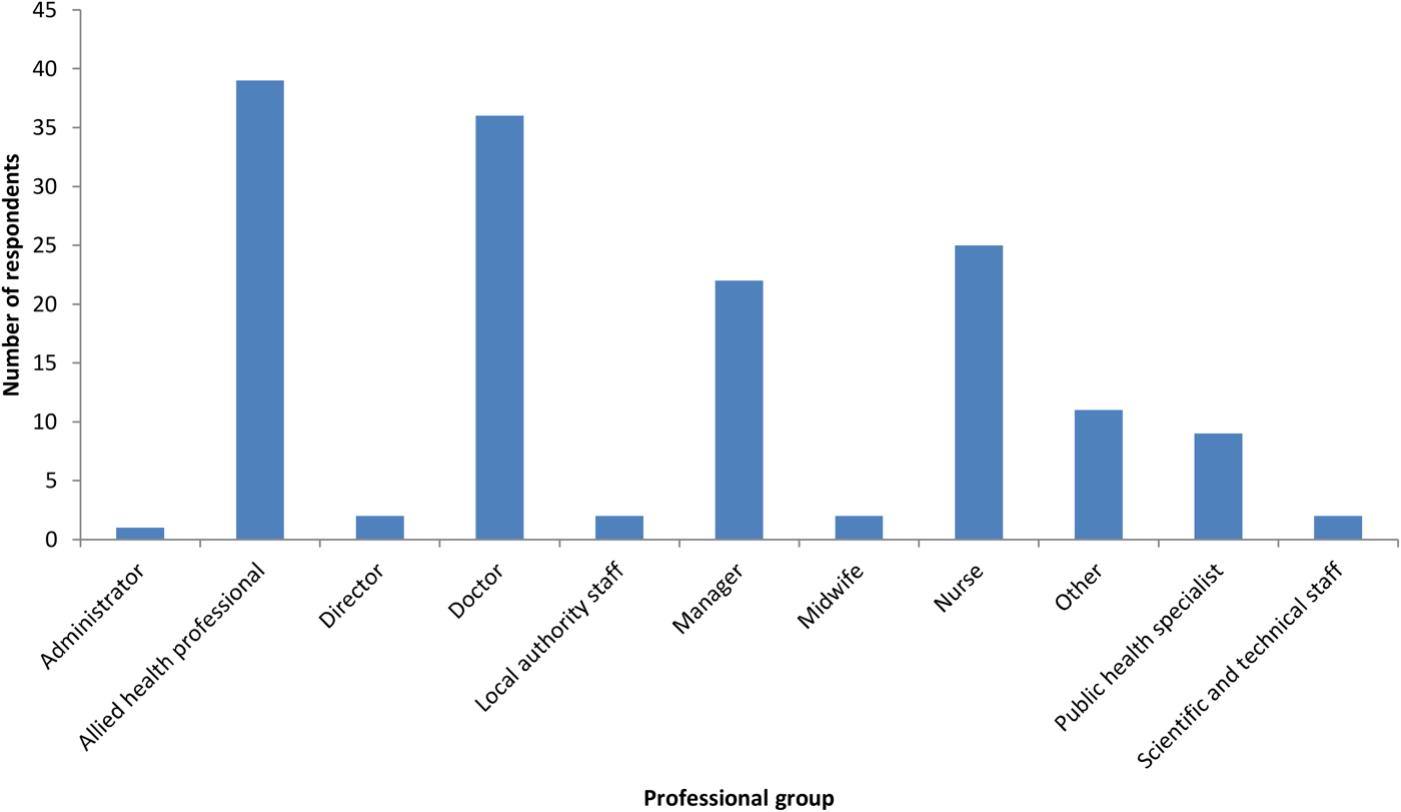
Professional groups represented by respondents.

Respondents were from 20 of the 35 CLAHRC healthcare and public health partner organisations. This included a wide spread of different types of organisation: 14 NHS provider organisations, including 4 teaching hospitals, 4 specialist mental health organisations and 1 community care provider; 3 local government public health departments; and 3 organisations responsible for commissioning or purchasing care on behalf of a geographical population. The median number of responses per organisation was 4.5 (IQR 1–7.25). As table 2 shows, over 50% (n=105) of survey respondents were staff in teaching or specialist hospitals. About 74.8% of respondents were women, 38.4% were aged 30–39 years and 33.1% aged 40–49 years. Respondents’ professions are outlined in figure 1. The largest four groups were allied health professionals (AHPs, 25.8% of sample); doctors (23.8%); managers (14.6%) and nurses (16.6%). Other groups included administrators (0.7%), directors (1.3%), local authority/public health staff (7.3%) and scientific/technical staff (1.3%).

**Table 2:** Types of health organisation represented

	Number of organisations	Total number of respondents
Teaching hospital	4	105
District general hospital	5	31
Mental/community health provider	5	47
Local government public health department	3	15
Clinical commissioning group	3	5
Total	20	203

The training needs and priorities of the whole survey sample are described below. We also highlight key differences between the four largest staff groups. The views of other staff groups are not described in detail, because the relatively small number of responses increases the risk that the data are not necessarily representative.

### Research training needs

In this section, we first describe participants’ self-assessment of the importance of the 25 tasks included in the questionnaire. We then compare this with the performance ratings assigned to each task, in order to assess research training needs. Information about the relative importance and performance of each task is provided in table 1 and data illustrating the distribution of responses is provided in online supplementary file 1.

10.1136/bmjopen-2016-012557.supp1 supplementary data

### Importance of tasks

Across the study population, participants rated the following as the most important tasks: managing multiple demands on your time (median score=7); teaching colleagues and/or students; evaluating your organisation's performance; making do with limited resources; and coping with change in the health service (median scores all=6). Of the 13 research-related tasks, participants considered applying research results to practice and accessing relevant research literature to be the most important (median scores both=6).

Designing research studies; accessing resources to undertake research (eg, money, information, equipment); securing time to undertake research; identifying viable research topics and statistically analysing your own research data were the three least important research tasks for study participants. Nevertheless, apart from designing research studies, each of these tasks had a median importance score of more than 4.0 on the seven-point scale, suggesting that these tasks are still considered relatively important to the respondents’ jobs. In addition, around 20% of respondents gave each of these tasks an importance score of 7.0 (‘very important’), which indicates that they are highly relevant to a subset of participants. Indeed, with the exception of designing research studies, all the 25 tasks included in the survey had a median importance score of more than 4.0, with 10 having a median score of 6.0 or more on the seven-point scale (table 1).

There were minor differences between the four largest professional groups in terms of the tasks identified as most important. Applying research results to practice; learning about new research developments and accessing relevant research literature were regarded as most important by doctors and AHPs. On the other hand, nurses and managers selected managing multiple demands on your time as one of the tasks most important to their role; introducing new ideas at work and evaluating organisational performance were also important to both these groups.

In line with the results for the whole study population, designing research studies was regarded as one of the least important tasks by all four professional groups. Identifying viable research topics was considered less important by both doctors and nurses, while nurses and AHPs also considered accessing resources for research (eg, money, information, equipment) to be relatively unimportant. Finally, managers also rated working as a member of a research team and writing up the findings of research or audits as relatively less important to their particular role.

### Training needs

Comparing the median self-assessed importance and performance rating for each task across the whole study population, we identified significant training needs for 24/25 tasks (p≤0.05; table 1). The only task without a significant difference between median importance and performance was undertaking administrative activities (p=0.15). Using this approach, it is possible that a training need might be identified, that relates to a task of moderate or little importance to participants. However, as we have noted, all tasks received a median importance rating of 4 or above, with the exception of designing research studies. Although a training need was identified for this task (p<0.01), the median importance score was only 3.0 on the seven-point scale.

Training needs were identified as those with a statistically significant difference between importance and performance scores. Across the study population, the three tasks with the largest training needs were managing multiple demands on time; learning about new research developments; and assessing the relevance of research. Other research-related tasks with large training needs were applying research to practice; securing time to undertake research; and accessing research literature.

Table 1 also compares the most significant training needs across the four largest professional groups. Managing multiple demands on time represented a key gap, and one of the most significant training needs, for all four groups. With regard to using and conducting research, learning about new research developments was also a key training need. In addition, AHPs particularly highlighted a need for training in applying research in practice, while evaluation was a key gap for managers.

### Research training priorities

**Figure 2:**
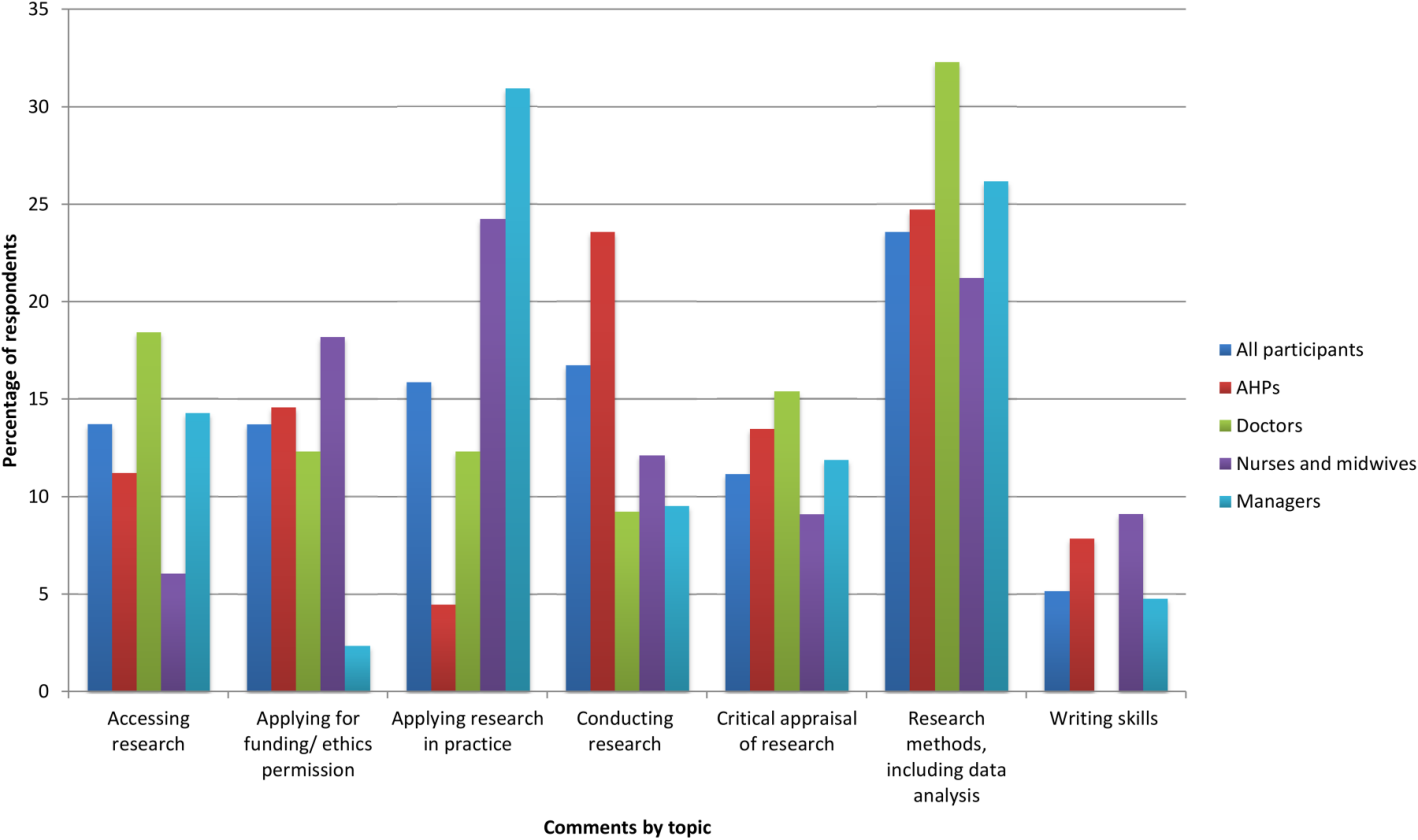
Research training priorities by professional group. AHPs, allied health professionals.

In the second section of the survey, participants were invited to list up to three research-related priority areas in which they would like to receive further training. Of the 203 participants 125 listed at least one priority. In total, we received 302 suggestions, which fell into eight categories (figure 2).

Considering the study population as a whole, the largest number of suggestions (n=55 suggestions) related to training in research methods, including data analysis. Indeed almost half of the priorities identified in this category (n=25) were for training in conducting and interpreting statistical analyses. Other participants sought training in techniques such as systematic reviewing or questionnaire development, while some also wanted to learn how to choose the most appropriate research design or method for a given project.

The practicalities of conducting research represented another training priority for participants (n=39 suggestions). Suggestions in this category included how to identify research topics and develop proposals, as well as guidance in aspects of the research process such as securing research ethics permissions. Further 32 comments related specifically to training in how to obtain funding to undertake research. Priorities here included how to identify and apply for appropriate sources of funding.

Another area of priority was finding and using research evidence in practice. Thirty-two of the 302 suggestions related to training in how to develop better online search strategies to identify relevant research quickly and effectively. We also received a further 26 suggestions about training in critical appraisal of research, while another 37 related to the process of applying research findings to practice. Priorities in this latter category included how to assess the relevance of research to a specific patient population or organisation, and how to use evidence in developing both business cases and clinical guidelines.

Figure 2 shows how the balance of priority areas differed between the four largest professional groups. Among doctors, the largest number of training suggestions related to research methods, including carrying out statistical analysis. This was also important to AHPs, along with training in the practicalities of conducting research. Nurses and managers also prioritised training in research methods, but the largest number of suggestions from both these groups related to training in how to apply research findings to change practice.

### Barriers to being involved in research

We did not directly ask participants about barriers that may impede them from using research or becoming involved in it. However, there are a range of factors beyond ‘pull’ efforts such as training to develop the capacity and capability of staff within health organisations.^9^ When asked to identify their priorities for training, a number of participants instead described challenges they face in using research or participating in it. As we have already noted, our findings highlight that many participants are faced with a lack of institutional support for them participating in research, including competing demands on their time, as well as dwindling resources. Some participants wanted a protected time slot each week to work on audits and research, or even research-related tasks, such as writing letters to journals. Most of these suggestions were made by doctors: some had previously had time allocated to undertake research, but found this later withdrawn because of a lack of funds.

A second key barrier was access to relevant equipment and resources. This particularly related to online publications. Participants sought ‘open access to all applicable research,’ ‘more access to online databases’ and ‘access to the university’s online library of journals’. More fundamentally, others reported that they did not have ‘access to a computer in the library for research’.

## Discussion

In recent years there has been recognition that there needs to be a marked shift from a supply-driven culture of research production, towards a more demand-driven approach, which seeks to foster a culture of partnership between academics and decisionmakers.^28^ ^29^ Within this, staff in healthcare and public health organisations have a key role to play,^12^ for example, within the NIHR CLAHRCs in England.^13^ ^15^ Such collaboration should involve coproducing AHR, and also improving patient care and population health through the implementation of its findings.^2^ Training is one of a range of factors which may help to facilitate access, dissemination, exchange and use of evidence within health organisations In this paper, we have described the findings of an exercise we conducted to inform our efforts to increase research use and participation across a large research collaboration through training, and in doing so better link research and action.^9^ We sought the views of a wide range of healthcare and public health staff about the training they need to conduct research and apply its findings in practice. To do this, we asked participants to self-assess the importance of tasks to their current role, as well as their current performance in carrying them out. Training needs were identified by comparing the mean of the two scores. Across the study population, two of the three research-related tasks with the largest training needs related to using research in practice: learning about new research developments and assessing the relevance of research. Key research training needs identified were similar across the four main professional groups. In contrast, however, in the free-text section of the questionnaire, the training priorities that participants described related not just to using research, but also to carrying it out. Priorities included training in research methods, including data analysis; study design and data collection; and applying research in practice. Accessing research evidence and applying for funding were also important. The balance of suggestions was similar for doctors, AHPs and nurses. However, conducting empirical research was less of a priority for managers, who focused more on the skills needed to use research findings. These results are perhaps not surprising. As Walshe and Rundall note, many clinicians receive some research methods training as part of their professional development. In contrast, managers often have no research training and the managerial culture is intensely pragmatic, valuing the application of ideas in practice more than it does in the search for knowledge about those ideas.^30^ It is, however, encouraging that managers in our survey highlighted a need for training in using research to inform their practice. Again compared with clinicians, personal experience and self-generated knowledge typically play a much larger part in determining how managers approach their jobs, and there is much less reliance on a shared body of formal knowledge in decision-making.^30^ Indeed, in the past it has been argued that much of the AHR evidence base lacks relevance to managers.^31^

Comparing the two exercises, accessing research and assessing its relevance emerge as key areas of need, across the study population, including the four largest population groups. The emphasis placed on conducting empirical research in the priority exercise suggests that there may also be demand for training in this area, although there was less emphasis on this in the importance/performance exercise. As we have highlighted, this also appears to be less relevant to managers, compared with doctors, AHPs and nurses. However, we did not collect detailed information on the seniority or authority of respondents, partly to protect their anonymity. This may though impact the ways in which participants respond. For example, those who consider service evaluation more important may be more senior. Participants across the study population also identified key gaps in managing multiple demands on time and securing time to undertake research, and highlighted a number of other challenges they face, including lack of time for research and lack of infrastructure, such as access to online publications. These need to be taken into account, as they may act as further barriers to research use and participation, potentially reducing the impact of training. However, these findings are based on participants' own self-assessment of the importance of each task to their current role, rather than an objective assessment of what is required of them, for example, in a job description. Therefore, there may be a discrepancy between what participants consider to be important, versus what their employers require of them. Nevertheless several professional groups were under-represented in our survey, and their perspectives warrant further exploration, for example, midwives and public health staff. In England, it is particularly important that we understand how the latter group might best be supported, following their transition from the NHS to local government.^32^ We also did not study primary care staff. Although participants represented a good spread of organisations, our sample size was relatively small. However, the survey was conducted in the first few months of our 5-year collaboration and it is encouraging to see that there was clear interest in using and applying AHR, right across the partnership. There are a number of limitations associated with using a convenience sample, not least because the results are of unknown generalisability.^33^ Staff who completed the survey are likely to be those who are most interested in the topic.^20^ ^22^ It is perhaps therefore not a surprise that most thought research-related tasks were important, especially given that 51% of responses were received from staff working in teaching or specialist hospitals. However, this sampling approach was appropriate for our purposes as we sought to reach those most interested in research, to prioritise our capability building efforts on this group in the first instance. We received responses from 20 of 35 CLAHRC partner healthcare or public health organisations. Indeed, the interest and training needs identified in the survey were consistent with our subsequent experience of running training events aimed at building capacity for healthcare and public health staff to use research and work with researchers. Demand has consistently exceeded supply and all events have been oversubscribed.

There is a range of literature exploring current research use and participation in specific groups, such as nurses^18^ and allied health professionals.^19^ Our study goes beyond this to examine self-identified opportunities to improve research use, as well as comparing across professional groups. Provider organisations have typically been under-represented in other surveys.^34^ In contrast, we looked across a range of different types of organisations, including providers of acute, mental health and community care. Our study adds to a growing body of literature exploring research training needs, and our findings align with what others have observed.^34^ However, this is the first such study to be conducted in England. We surveyed staff across a large research partnership, and received responses from 20 separate organisations, which ranged from large teaching hospitals, to small district general hospitals, as well as public health organisations. Our findings may therefore be relevant to others who are looking to establish similar training programmes. Nevertheless, training is only one of a range of factors which may help to facilitate use of and participation in research. A plethora of challenges and barriers can also be present at various levels within a health system, including ensuring ‘buy-in’ from upper management and lack of appropriate infrastructure. Owing to the range of potential challenges, interventions should be considered within the context of wider systems issues.^8^

Although our quantitative approach enabled us to seek input from staff from a broad range of organisations, it provided little opportunity for us to understand the complexity of responses. For example, we are aware that a number of organisations represented in the survey provide training and support for staff in finding and appraising research, via their library services. However, this was identified as one of the most significant training needs in both parts of this survey. It is not clear why existing training provision is not meeting this need. There is also a need to further explore the optimal ways to delivery training of this kind, perhaps using qualitative methods and how this might link in with the literature on barriers to research use.^6^ Finally, there is still only a limited literature on the long-term outcomes and effectiveness of different training opportunities, including how research use might be sustained in the longer term.^34^

The need to speed up the translation of research into practice is a priority for researchers and funding bodies, alongside efforts to promote the coproduction of knowledge. In this study, we describe the areas where healthcare and public health staff may benefit from further training in using and doing applied health research, to better link research and action.^9^ These include accessing research and assessing its relevance, as well as the skills required to carry out empirical research, such as data analysis. The priority study participants placed on all these topics, suggests that there would be demand for training if it were provided. Learning opportunities addressing these needs may help to improve the diffusion and adoption of research findings, and hence the quality of healthcare and public health services, for the benefit of patients and populations.
