# Supplementary material for: Building capacity to use and undertake research in health organisations: a survey of training needs and priorities among staff
Source: BMJ Open. 2016 Dec 7;6(12):e012557. doi: 10.1136/bmjopen-2016-012557 (PMC5168601; doi:10.1136/bmjopen-2016-012557)
Supplement: supplementary data [file bmjopen-2016-012557supp.pdf]

**Supplementary Information: Distribution of overall importance and performance scores for questionnaire tasks**

| Task | Importance scores | | Performance scores | |
|---|---|---|---|---|
| | Median | Interquartile range | Median | Interquartile range |
| 1. Handling routine data | 6 | 5-7 | 5 | 5-7 |
| 2. Critically evaluating published research | 5 | 4-7 | 4 | 3-5 |
| 3. Evaluating your organisation's performance | 6 | 4-7 | 4 | 3-5 |
| 4. Interpreting research findings | 5 | 4-7 | 5 | 4-5 |
| 5. Applying research results to your own practice | 6 | 5-7 | 4 | 4-5 |
| 6. Identifying viable research topics | 4 | 3-5.75 | 3 | 2-5 |
| 7. Introducing new ideas at work | 6 | 5-7 | 5 | 4-5 |
| 8. Accessing relevant research literature to inform your work | 6 | 5-7 | 5 | 3-6 |
| 9. Giving information about research to patients/the public | 5 | 4-6 | 4 | 3-5 |
| 10. Statistically analysing your own research data | 4 | 2-6 | 3 | 2-5 |
| 11. Teaching colleagues and/or students | 6 | 5-7 | 5 | 5-6 |

| | | | | |
|---|---|---|---|---|
| 12. Managing multiple demands on your time | 7 | 6-7 | 5 | 4-6 |
| 13. Writing up the findings of research studies or audits | 5 | 3-7 | 4 | 3-5 |
| 14. Undertaking health promotion activities | 5 | 3-6 | 4 | 2-5 |
| 15. Making do with limited resources | 6 | 5-7 | 5 | 4-6 |
| 16. Assessing local health care needs | 5 | 3-6 | 4 | 2-5 |
| 17. Collecting and collating relevant research | 5 | 3-6 | 4 | 3-5 |
| 18. Designing research studies | 3 | 2-6 | 3 | 2-4 |
| 19. Working as a member of a team doing research | 4 | 2-6 | 4 | 3-5 |
| 20. Accessing resources to undertake research e.g. money, information, equipment | 4 | 2-6 | 2 | 1-4 |
| 21. Undertaking administrative activities | 5 | 4-6 | 5 | 4-6 |
| 22. Personally coping with change in the health service | 6 | 5-7 | 5 | 4-5 |
| 23. Securing time to undertake research | 5 | 2.5-7 | 2 | 1-4 |
| 24. Learning about new research developments in your field | 6 | 5-7 | 4 | 3-5 |
| 25. Assessing the relevance of research to your organisation | 5 | 4-7 | 4 | 3-5 |
